# Chromodomain helicase DNA binding protein 5 plays a tumor suppressor role in human breast cancer

**DOI:** 10.1186/bcr3182

**Published:** 2012-05-08

**Authors:** Xiao Wu, Zhengmao Zhu, Weidong Li, Xiaoying Fu, Dan Su, Liya Fu, Zhiqian Zhang, Ang Luo, Xiaodong Sun, Li Fu, Jin-Tang Dong

**Affiliations:** 1 Department of Genetics and Cell Biology, Nankai University College of Life Sciences, 94 Weijin Road, Tianjin 300071, China; 2 Department of Hematology and Medical Oncology, School of Medicine; Winship Cancer Institute, Emory University, 1365 Clifton Road, Atlanta, GA 30322, USA; 3 Key Laboratory of Breast Cancer Research, Department of Breast Cancer Pathology and Research Laboratory, Cancer Hospital of Tianjin Medical University, Huan Hu Xi Road, Tianjin 300060, China

## Abstract

**Introduction:**

The chromodomain helicase DNA binding protein 5 (CHD5) has recently been identified as a tumor suppressor in a mouse model. The *CHD5 *locus at 1*p*36 is deleted, and its mutation has been detected in breast cancer. We, therefore, evaluated whether *CHD5 *plays a role in human breast cancer.

**Methods:**

We screened mutations in 55 tumors, determined promoter methylation in 39 tumors, measured RNA expression in 90 tumors, analyzed protein expression in 289 tumors, and correlated expression changes with clinicopathological characteristics of breast cancer. Functional effects of CHD5 on cell proliferation, invasion and tumorigenesis were also tested.

**Results:**

Although only one mutation was detected, *CHD5 *mRNA expression was significantly reduced, accompanied by frequent genomic deletion and promoter methylation, in breast cancer. The extent of methylation was significantly associated with reduced mRNA expression, and demethylating treatment restored *CHD5 *expression. Lower *CHD5 *mRNA levels correlated with lymph node metastasis (*P *= 0.026). CHD5 protein expression was also reduced in breast cancer, and lack of CHD5 expression significantly correlated with higher tumor stage, ER/PR-negativity, HER2 positivity, distant metastasis and worse patient survival (*P *≤ 0.01). Functionally, ectopic expression of *CHD5 *in breast cancer cells inhibited cell proliferation and invasion *in vitro *and tumorigenesis in nude mice. Consistent with the inhibition of invasion, CHD5 down-regulated mesenchymal markers vimentin, N-cadherin and ZEB1 in breast cancer cells.

**Conclusion:**

Down-regulation of CHD5, mediated at least in part by promoter methylation, contributes to the development and progression of human breast cancer.

## Introduction

Tumorigenesis is a multi-step process that results from progressive accumulation of genetic and epigenetic alterations in different genes. Chromosomal loss, which leads to the inactivation of tumor suppressor genes, is one of the most common genetic alterations detected in human cancer. Previous publications have documented that the p36 band of chromosome 1, 1*p*36, is frequently deleted in a wide range of human cancers, including those of epithelial, neural and hematopoietic origin [1]. Recently, Bagchi *et al. *identified *CHD5*, which localizes to the deletion region at 1*p*36, as a tumor suppressor gene through functional analysis in a mouse model. *Chd5 *knock-down was associated with hyperproliferation and reduced apoptosis and senescence, primarily through the p19^Arf^/p53 pathway [2].

CHD5 belongs to the chromodomain helicase DNA binding domain (CHD) family, which is a subclass of the Swi/Snf proteins [3,4]. Of nine members of the CHD family (CHD1-CHD9), two (CHD3 and CHD4) are components of the nucleosome remodeling and histone deacetylation (NuRD) complex and play an important role in chromatin remodeling [5,6]. As CHD5 shares the same functional domain with CHD3 and CHD4, it may also modulate chromatin remodeling and thus affect normal development and cancer.

Evidence that CHD5 functions as a tumor suppressor in human cancer has principally come from studies of neuroblastoma, in which *CHD5 *mRNA expression was down-regulated likely through promoter methylation in tumors, and high expression of CHD5 was statistically associated with better patient survival [7]. Furthermore, ectopic expression of CHD5 in neuroblastoma cell lines suppressed clonogenicity and tumor growth [6,7]. Hypermethylation of *CHD5 *promoter has also been detected in gastric, colorectal and ovarian cancers, and somatic mutations have been detected in ovarian cancer [8-11]. However, whereas 1*p*36 is commonly deleted in human breast cancers, the role of CHD5 in breast cancer has not been evaluated.

We hypothesize that *CHD5 *is a tumor suppressor gene in breast cancer and tested this hypothesis in this study. We examined *CHD5 *for somatic mutation, copy number changes, mRNA and protein expression, and promoter methylation in primary tumors and cell lines from human breast cancer. In addition, we assessed its effect on cell proliferation *in vitro *and *in vivo*. We found that while *CHD5 *mutation was relatively rare, it had frequent down-regulation, hemizygous deletion and promoter methylation. Promoter hypermethylation correlated with lower levels of *CHD5 *mRNA expression, and demethylating treatment decreased promoter methylation and increased *CHD5 *expression, suggesting that promoter methylation is responsible at least in part for reduced *CHD5 *expression in breast cancer. Interestingly, reduced *CHD5 *expression significantly correlated with lymph node metastasis, recurrence and shorter patient survival in breast cancer. In addition, ectopic expression of *CHD5 *suppressed cell proliferation and tumor growth of the MDA-MB-231 cell line by arresting cell cycle at the G0/G1 phase, and inhibited invasiveness of MDA-MB-231 and Hs 578T cells *in vitro*. Our results strongly support a tumor suppressor role for *CHD5 *in breast cancer.

## Materials and methods

### Cell lines and tissues

In total, 32 breast cancer cell lines (BT-20, BT-474, BT-549, BT-483, CAMA-1, DU4475, HCC1143, HCC1395, HCC1500, HCC1599, HCC1806, HCC1937, HCC202, HCC2218, HCC38, HCC70, Hs 578T, MCF7, MDA-MB-134, MDA-MB-157, MDA-MB-175, T-47D, MDA-MB-231, MDA-MB-361, MDA-MB-415, MDA-MB-453, MDA-MB-468, SW527, UACC893, ZR-75-1, ZR-75-30, BRF-71T) and two immortalized non-neoplastic breast epithelial cell lines (184A1 and BRF-97T) were used in this study. Of these, BRF-97T and BRF-71T were purchased from Biological Research Faculty & Facility (BRFF, Ijamsville, MD, USA), and the rest were purchased from American Type Culture Collection (Manassas, VA, USA). The primary culture of human mammary epithelial cells (HMEC) was purchased from Cambrex (East Rutherford, NJ, USA). Cells were cultured according to the manufacturers' protocols.

Tumor tissues and adjacent normal tissues were obtained from 377 breast cancer patients who received surgery in the Cancer Hospital of Tianjin Medical University, Tianjin, China. Freshly frozen tissues from 107 patients were used to extract RNA or DNA samples, of which 58 were stored in RNAlater^® ^solution and used for RNA extraction and *CHD5 *mRNA expression analysis, 30 had bisulfite-treated DNA from a previous study and were used for promoter methylation analysis (19 of them also used for RNA expression analysis), and 38 were used for mutation detection. Formalin-fixed paraffin-embedded primary tumor specimens from 289 patients were prepared in a tissue microarray format and used for CHD5 protein expression analysis by immunohistochemical (IHC) staining, which included 19 tumor specimens that were also used for mRNA expression and promoter methylation analyses. Samples were from the tissue bank at the Cancer Hospital of Tianjin Medical University and were used entirely based on availability. Use of human tissues and clinicopathological information was approved by the Institutional Ethics Committee. Genomic DNA was extracted using the DNeasy Blood & Tissue kit (Qiagen, Shanghai, China ), and total RNA was isolated using the RNeasy Mini kit (Qiagen).

The numbers of samples for different analyses were different because we used samples from our previous studies and many of them ran out of DNA, RNA or bisulfite-treated DNA. The majority of specimens used for IHC staining did not have DNA or RNA samples available. All samples with a result for any readout were included in this manuscript.

### Real-time and semi-quantification PT-PCR

Reverse transcription reaction was performed using 2 μg total RNA with M-MLV reverse transcriptase (Promega, Madison, WI, USA). Real-time PCR reaction was performed in 25 μl reaction volume using SYBR *Premix Ex Taq *kit (TaKaRa, Dalian, China) and carried out on an iQ5 Multicolor Real-Time PCR Detection System (Bio-Rad, Hercules, CA, USA). The *CHD5 *expression levels were normalized according to *GAPDH *in each sample. Primers used for *CHD5 *real time PCR were 5'-CAAGTGTAAAGGGAAGCGGAAGAAG-3' (forward) and 5'-CTTTTTATTCGGGGAGTAGTCAC-3' (reverse), and those for *GAPDH *were 5'-GGTGGTCTCCTCTGACTTCAACA-3' (forward) and 5'-GTTGCTGTAGCCAAATTCGTTGT-3' (reverse). *CHD5 *mRNA expression reading in HMEC was defined as 1, and *CHD5 *expression levels for other samples were normalized accordingly. Primer sequences for *CHD5 *semi-quantification PCR were 5'-TGAAGAAACTGCGGGATG-3' (forward) and 5'-TGCCGAACTTGAGGATGT-3' (reverse).

### Mutation and methylation analysis

The 40 coding exons and adjacent splicing junctions of *CHD5 *were amplified by PCR using previously reported primers and procedures [12]. PCR products were purified and sequenced (Invitrogen, Beijing, China). When a sequence alteration was detected, PCR and sequencing were repeated to confirm it. For confirmed alterations in tumor samples, PCR and sequencing were performed in matched normal samples to determine whether an alteration was somatic or germline.

Promoter methylation analysis was conducted following a published method [13]. Two pairs of primers, 5'- TTTAGGGGTTTGTATGGGTTTTAG-3' (F1)/5'-CCCTCTCCAAAAAAAATTAAAAAA-3' (R1) [7] and 5'-TTTTTTTGGAGAGGGGGTTAGG-3' (F2)/5'-CTATAACAACCCCATCCCAT-3' (R2), were used. These primers amplify the CG rich region of the *CHD5 *promoter from -841 to -246. Purified PCR products were cloned into pMD18-T vector (TaKaRa, Dalian, China). At least eight clones for each sample were sequenced. Methylation levels for each CpG site were indicated by the ratio of methylation positive clones to the total number of clones sequenced, and were categorized into three groups in cell lines: high (ratio > 0.5), low (0 < ratio < 0.5) and none (ratio = 0) (marked as dark, gray and white dots, respectively.). The average methylation level at a CpG site was also presented.

### Copy number detection

Copy number changes in the *CHD5 *gene were detected by real-time PCR using the SYBR *Premix Ex Taq *kit (TaKaRa). Primer sequences for *CHD5 *were 5'-CTGTTGCTGCAGTTCCTTCTC-3' and 5'-ATGAAGGACAGAACCTGCCTG-3'. The *RNaseP *gene, which rarely has copy number changes in tumors [14], was used as an internal control for a stable diploid copy number. Primer sequences for *RNaseP *were 5'-CCTGGTACATGCCACTGATG-3' and 5'-AGTGTAGAGGGCAAGCCAGA-3'. Briefly, 10 ng genomic DNA from each sample was used as the template for PCR with either *CHD5 *or *RNaseP *primers in a volume of 20 μl. The final concentration for each primer was 0.25 μM. *CHD5 *copy number was determined by dividing the ΔCt value for *CHD5 *by that for *RNaseP*. For cell lines, the average *CHD5*/*RNaseP *ratio among a pool of normal human genomic DNA (HGD) was defined as 1 and used as the value for a normal genome, and a deletion was defined by a ratio that was 0.5 or less. For primary tumors, a deletion was defined when the *CHD5*/*RNaseP *ratio in a tumor was about half or less of that in its matched non-cancerous tissue.

### Western blotting

Western blotting was performed according to a published protocol [15]. Antibodies for the following proteins were used: CHD5 (rabbit polyclonal, 1:1,000 dilution, Strategic Diagnostics, Newark, DE, USA), β-actin (A1978, Sigma-Aldrich, Beijing, China), p21 (#610233, BD Biosciences, Franklin Lakes, NJ, USA), p53 (sc-47698, Santa Cruz Biotechnology, Santa Cruz, CA, USA), vimentin (#3932, Cell Signaling Technology, Beverly, MA, USA), N-cadherin (#610920, BD Biosciences), E-cadherin (#3195, Cell Signaling) and ZEB1 (sc-25388, Santa Cruz Biotechnology).

### Immunohistochemical (IHC) staining

Immunohistochemical analysis was performed as previously described [16], using polyclonal rabbit anti-CHD5 antibody (1:2,000 dilution). The pre-immune serum was used as the negative control. In evaluating the specificity of the CHD5 antibody, we made cell pellets of ZR-75-1 (CHD5-negative) and T-47D (CHD5-positive) cells in agarose, fixed them in formalin, embedded in paraffin, prepared sections, and then conducted IHC staining. Detection of strong signals in T-47D but no signals in ZR-75-1 cells validated the antibody for IHC staining.

Protein expression levels of CHD5 were calculated by multiplying the intensity of nucleus staining (0 = no staining, 1 = low intensity, 2 = medium intensity, and 3 = high intensity) with the percentage of positively stained cells (0 = no, 1 = 1 to 25%, 2 = 26 to 50%, 3 = 51 to 100%). A combined index (intensity score × percentage score) of 0 or 1 was considered as negative staining (-), while an index of 2 or 3 as weak staining (+), 4 to 6 as moderate staining (++), and 9 as strong staining (+++). In statistical analysis, CHD5 expression levels were categorized into two groups: lack of expression (-) and weak to strong expression (+, ++, and +++).

### Colony formation assay

The *CHD5 *expression plasmid was constructed by cloning the coding region of *CHD5 *into the pcDNA3.0 vector (Invitrogen). MDA-MB-231 cells were seeded in 6-well plates at a density of 5 × 10^5 ^cells per well and transfected by using the lipofectamine 2000 reagent (Invitrogen, Beijing, China). Forty-eight hours after transfection, one well of cells was harvested to confirm CHD5 expression, and the remaining wells were cultured in selection medium containing 800 μg/ml G418 (Sigma) for two weeks. Cells were then fixed, stained with sulforhodamine B (SRB), and measured for optical intensities, which indicated cell numbers, as described in our previous publication [17].

### RNA interfering

Small-interfering RNAs (siRNAs) for the *CHD5 *gene were purchased from Qiagen and Invitrogen. Two of them, named Q7 (Qiagen) and I20 (Invitrogen), were confirmed to be capable of knocking down *CHD5 *and thus were used. The target sequence of Q7 was 5'-ACGGTACATGATCCTCAACGA-3' and that of I20 was 5'-CAGCAGTTCTGCTTCCTCCTCTCAA-3'. A siRNA that does not target any known genes was purchased from Rui Bo (Guangzhou, China) and used as a control. The Lipofectamine RNAiMAX transfection reagent (Invitrogen) was used to transfect siRNAs into cells following the manufacturer's instruction.

### Flow cytometric cell cycle analysis

MDA-MB-231 cells transfected with pcDNA3.0 vector or *CHD5 *expression plasmid were selected using G418 (800 μg/ml; Sigma) for two weeks. 1 × 10^6 ^cells of each group were collected, fixed with 70% ethanol for 24 hours, washed and re-suspended with phosphate-buffered saline (PBS), incubated with propidium iodide (20 mg/ml, Sigma) and Rnase A (20 mg/ml) for 30 min in the dark, and analyzed on a BD FACSCalibur flow cytometer (Franklin Lakes, NJ, USA).

### Cell proliferation analysis

After transfection and selection with G418 for two weeks, MDA-MB-231 cells were harvested, diluted into five cells/ml using selection medium, plated into 96-well plates at 100 μl per well, and cultured for another two to three weeks. Cells surviving in a well were propagated and confirmed for *CHD5 *expression. Parental or clones of transfected cells were plated into 12-well plates at 3 × 10^4 ^cells/well, cultured for different times, fixed, stained with SRB and measured for cell numbers.

Hs 578T cells were also transfected with CHD5-expressing plasmid or control plasmids using lipofectamine 2000 reagent. After selection using G418 (400 μg/ml) for two weeks, the population cells of each group were plated into 12-well plates and used for cell proliferation analysis.

### Tumor growth assay

One × 10^6 ^parental or clones of transfected MDA-MB-231cells were mixed with Matrigel (BD Biosciences, San Jose, CA, USA) and injected subcutaneously into four weeks old female BALB/c nude mice (eight mice for each group). Tumor volumes were measured once a week and calculated as below: L × W^2 ^× 0.523 mm^3^, where L and W indicate the length and the width of a tumor. Mice were sacrificed at five weeks after injection, and tumors were surgically removed, weighed, and fixed in 4% formalin. Hematoxylin and eosin staining was performed for histological examination. The animal experiment was approved by the animal care and use center of Nankai University, Tianjin, China.

### Invasion assay

The Boyden chamber assay was used for invasion assay. Briefly, 1 × 10^5 ^cells suspended in 200 μl serum-free medium were plated into the top chamber with 50 μl Matrigel-coated membrane (8 μM pole size, BD Biosciences, Shanghai, China). The chambers were then placed into 24-well plates with 600 μl serum-containing (10%) medium in each well. After 24 hours incubation, cells on the bottom side of the chamber membrane were fixed, stained with crystal violet, and photographed.

### Statistics

The SPSS 15.0 software package (SPSS Inc, Chicago, IL, USA) was used for statistical analysis. Correlations between CHD5 expression and clinicopathological variables were evaluated by Chi-square (χ^2^) test. Progression-free survival (PFS) and overall survival (OS) rates were estimated by the Kaplan-Meier analysis. The Cox proportional hazard regression analysis was performed for the identification of relevant prognostic factors. For tumorigenesis experiments, tumor sizes or weights between two groups were analyzed using the Student's *t *test. The statistical analysis was two-sided and *P-*values less than 0.05 were considered as statistically significant.

## Results

### Mutation and copy number changes of *CHD5 *in breast cancer

Sequencing the coding exons and adjacent splicing junctions of *CHD5 *in 38 primary tumors and 17 cell lines of breast cancer identified only one heterozygous frameshift mutation (3215delG) in the MDA-MB-231 cell line. A novel single nucleotide polymorphism (SNP) (470C > T, Thr157Met) at the N-terminal region of *CHD5 *was detected in two tissue samples. In addition, eight known SNPs (previously reported in the SNP database) were detected in the primary tumors and/or cell lines. All sequence alterations, including known SNPs identified in this study, are summarized in Table 1.

**Table 1:** Mutation and single nucleotide polymorphisms (SNPs) of *CHD5 *in 55 breast cancer samples

Exon	Sequence alteration	Amino acid alteration	Allele frequency	
			
			Cell lines	Tissues
*Mutation *				
21	DelG3315	-	1/34	0/76
*SNPs*				
4*	C570T	Thr157Met	0/34	3/76
4	G529C	Leu	8/34	0/76
7	C1003T	Phe	15/34	55/76
8	A1204G	Val	16/34	55/76
12	C1957T	Tyr	3/34	2/76
15	C2479T	Asn	0/34	23/76
16	T2593C	Ile	11/34	46/76
22	G3436A	Ala	0/34	27/76
31	T4715C	Ser1539Pro	26/34	0/76

Notes: 17 cell lines and 38 primary tumors of breast cancer were used. * indicates the novel SNP identified in this study. The rest of the SNPs have been reported in the SNP database (dbSNPs).

**Figure 1:**
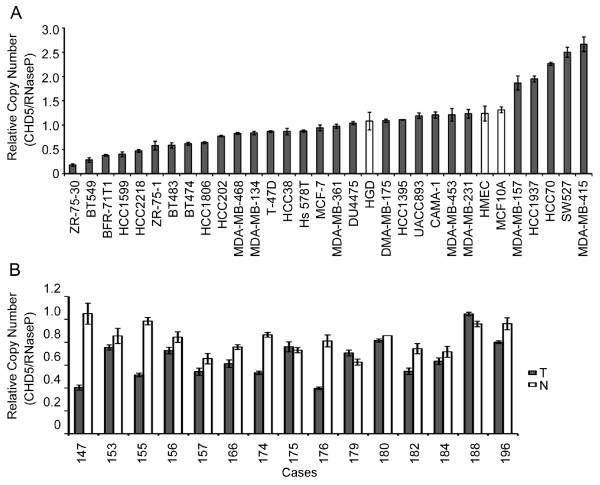
**Detection of *CHD5 *copy number changes in breast cancer**. A total of 29 cell lines (**A**) and 15 primary tumors with matched normal tissues (**B**) from breast cancer were used. A pool of normal human genome DNA was used as the normal control. Real-time PCR was used for the detection, with the *RNaseP *gene as an internal control that rarely has copy number changes in cancers. T, tumor tissue; N, normal breast tissue.

Changes in *CHD5*'s copy number were determined in 29 breast cancer cell lines and 15 paired breast cancer tissues by real-time PCR with a pool of normal human genomic DNA as the control for normal genome. Whereas no homozygous deletion was detected, 5 of the 29 (17%) cell lines and 3 of the 15 (20%) primary tumors showed a deletion in one to the two *CHD5 *copies. In addition, 5 of the 29 (17%) cancer cell lines had an increased copy number (Figure 1).

### Down-regulation of *CHD5 *in breast cancer

**Figure 2:**
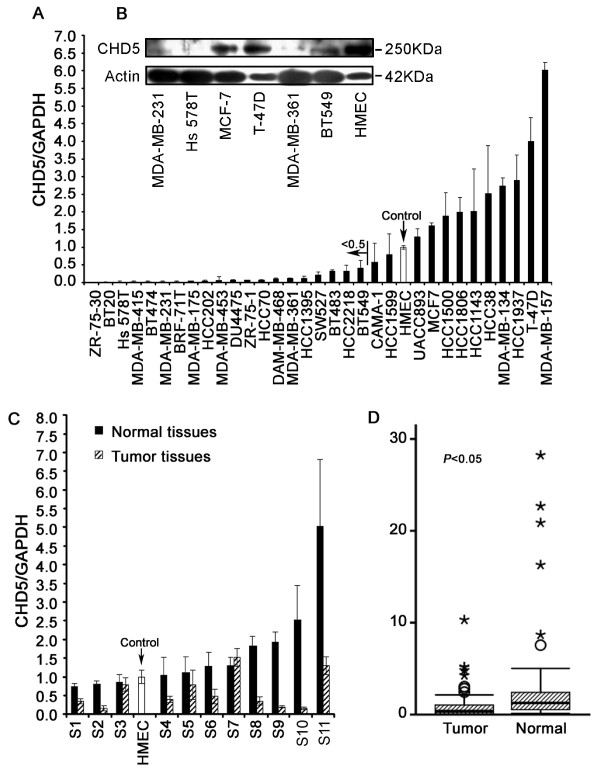
**Down-regulation of *CHD5 *mRNA expression in breast cancer**. (**A**) Expression of *CHD5 *mRNA in breast cancer cell lines and primary culture of human mammary epithelial cells (HMEC), as detected by real-time RT-PCR. *GAPDH *was used for normalization. The ratio *CHD5*/*GAPDH *in HMEC was defined as 1, and the ratio in other cell lines was adjusted accordingly. (**B**) Detection of CHD5 protein expression in six breast cancer cell lines and in HMEC cells by Western blotting. Actin was used as a loading control. (**C**) Representative results of *CHD5 *mRNA expression in breast cancer samples and matched normal tissues, as detected by real-time RT-PCR using HMEC as a positive control. The ratio of C*HD5*/*GAPDH *in HMEC was defined as 1, and that in other samples were normalized accordingly. (**D**) The distribution of *CHD5 *mRNA expression values in the tumor group (*n *= 58) versus that in the normal group (*n *= 58) are shown in the plot-box diagram. The box ranges from the 25^th ^to the 75^th ^percentile, and the bold line across the box indicates the median. The whiskers are 1.5 times of the quartile values.

*CHD5 *mRNA expression was determined in 32 cell lines and 58 primary tumors of breast cancer by real time PCR. Compared to HMEC, 20 of 32 cancer cell lines (62.5%) showed reduced expression of *CHD5 *by at least 50%, and 17 (53.1%) had an reduction by at least 75%. While six cell lines (18.8%) did not have obvious expression changes, the remaining six cell lines (18.8%) showed an increased expression of *CHD5 *by at least one fold (Figure 2A). CHD5 protein expression was confirmed in six breast cancer cell lines and HMEC cells by Western blotting. Consistent with real time PCR results in cell lines, CHD5 protein was strongly expressed in T-47D and HMEC, moderately expressed in BT549 and MCF-7, but not detectable in MDA-MB-361, MDA-MB-231 and Hs 578T (Figure 2B). All six, except the BT549 cell line, had a normal *CHD5 *copy number.

Compared to matched adjacent noncancerous tissues, 37 of 58 primary tumors (63.8%) showed a reduced *CHD5 *mRNA expression by at least 50%, 17 (29.3%) had little change, and 4 (6.9%) had an increased *CHD5 *expression. In 20 of the 58 (34.5%) tumors, *CHD5 *was down-regulated by at least 75%. Representative results from 11 paired samples are shown in Figure 2C. Statistical analysis demonstrated that *CHD5 *mRNA expression was significantly reduced in breast tumors compared to their matched normal tissues (independent *t*-test, *P *< 0.05, Figure 2D). Furthermore, reduced *CHD5 *mRNA expression significantly correlated with lymph node metastasis (Mann-Whitney U test, *P *< 0.05), but not with age at diagnosis or the status of p53, ER, PR and HER2 (Additional file 1 Table S1).

**Figure 3:**
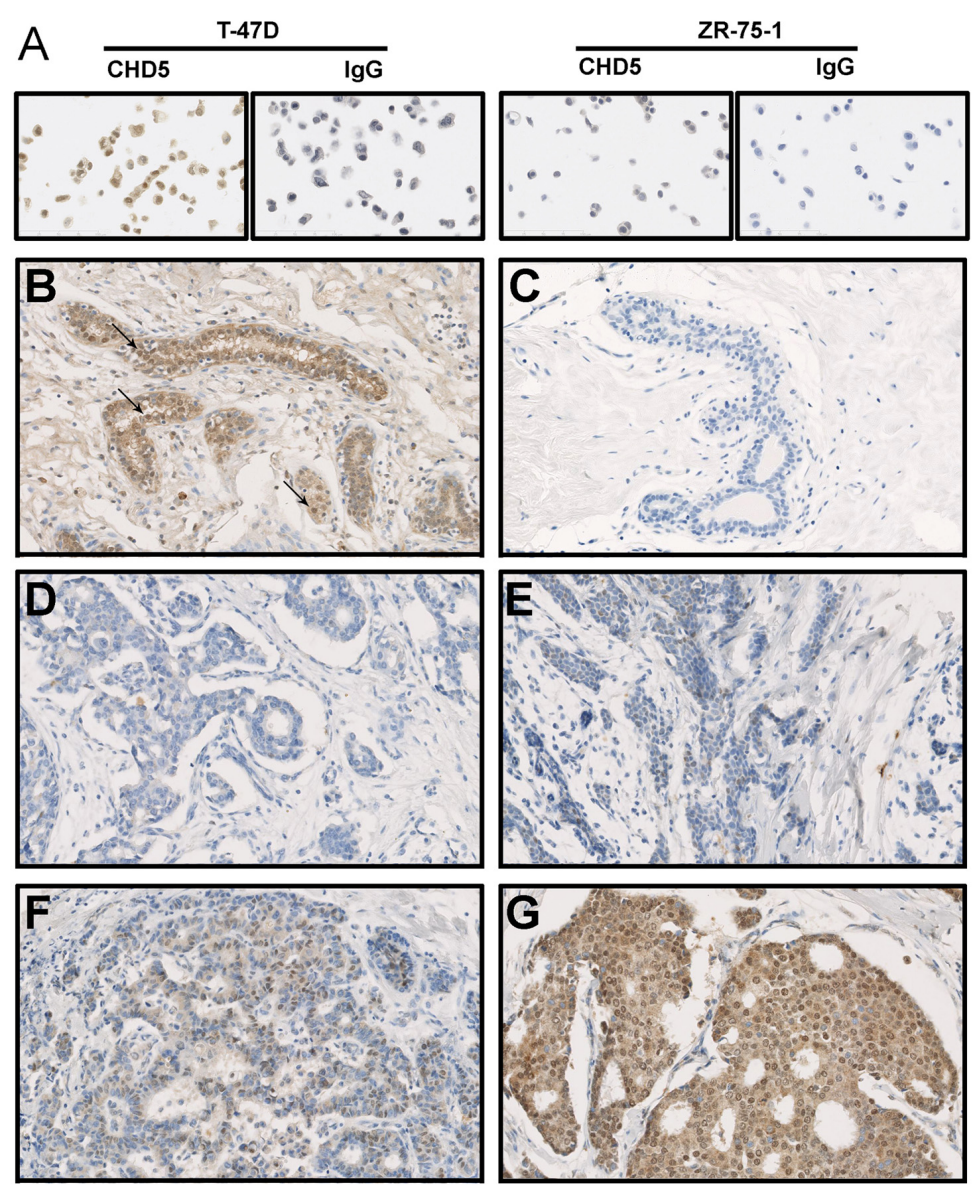
**Immunohistochemical detection of CHD5 protein in normal tissues and primary tumors of the human breast**. (**A**) Validation of CHD5 antibody by detecting CHD5 protein in CHD5-positive T-47D cells but not in CHD5-negative ZR-75-1 cells by IHC with the CHD5 antibody. Rabbit preimmune IgG was used as the negative control. (**B, C**) Normal breast tissue stained with rabbit anti-CHD5 antibody (B) or preimmune rabbit IgG (C, negative control). Arrows indicate cells that are positive for CHD5 staining. (**D-G**) Representative breast cancers showing no CHD5 protein expression (D) and low (E), medium (F) and high (G) levels of CHD5 expression.

CHD5 protein expression was examined in 289 formalin-fixed paraffin-embedded tumors and 20 normal breast tissues by IHC. We first examined the specificity of the CHD5 antibody in IHC staining (Figure 3A). CHD5 protein was positively identified in the nucleus of all 20 normal tissues (Figure 3B, Additional file 1 Table S2). In the breast cancer tissues, while 145 (50.2%) tumors showed various levels of CHD5 protein expression, 144 of them (49.8%) showed no obvious CHD5 expression (Figure 3D-G, Additional file 1 Table S2). Statistical analysis indicated that lack of CHD5 expression significantly corrected with higher histological grade (*P *= 0.01), more HER2 positive tumors (*P *< 0.001), and less ER- (*P *= 0.009) or PR-positive (*P *= 0.001) tumors (Table 2). According to molecular classification of breast cancer, more CHD5-positive breast cancers belonged to the luminal A subtype (ER+ and/or PR+, HER2-), while more CHD5-negative tumors belonged to the luminal B (ER+ and/or PR+, HER2+), HER2 (ER-, PR-, HER2+), or the triple negative subtype (ER-, PR-, HER2-) (*P *< 0.001). No association was detected between CHD5 expression and patient age at diagnosis, tumor size, lymph node metastasis, or family history (Table 2).

**Table 2:** Correlation between CHD5 expression and clinicopathological parameters in breast cancer

Parameters	CHD5 expression (*n*)		Total cases	*P-*values
	**-**	**+**		
Age (years)			289	0.590
≤ 50	67	62	129	
> 50	78	82	160	
Tumor size			288*	0.476
≤ 2 cm	29	34	63	
> 2 cm	115	110	225	
Histological grade			275*	0.010
I	4	14	18	
II	100	103	203	
III	34	20	54	
pTNM stage			284*	0.105
I/II	99	111	210	
III/IV	43	31	74	
LN metastasis			289	0.215
-	57	67	124	
+	88	77	165	
Estrogen receptor			289	0.009
-	66	44	110	
+	79	100	179	
Progesterone receptor			289	0.001
-	84	54	138	
+	61	90	151	
HER2 status			289	< 0.001
-	74	111	185	
+	71	33	104	
Molecular subtype			289	< 0.001
luminal A	54	95	149	
luminal B	35	12	47	
HER2	37	20	57	
Triple negative	19	17	36	
Family history			264*	0.420
No	105	98	203	
Yes	28	33	61	
Distant metastasis			289	< 0.001
No	115	137	252	
Yes	30	7	37	
Death			289	0.001
No	125	140	265	

Yes	20	4	24	

Notes: LN, lymph node; pTNM, pathological tumor node metastasis; *, analysis was limited to patients whose relevant information was available. Molecular subtypes were defined as below: luminal A, ER+ and/or PR+/HER2-; luminal B, ER+ and/or PR+/HER2+; HER2+, ER-/PR-/HER2+; and triple negative, ER-/PR-/HER2-. CHD5 expression is categorized into two groups: lack of expression "-" and expression at various levels "+". *P-*values were determined by the Chi-square (χ^2^) test.

**Figure 4:**
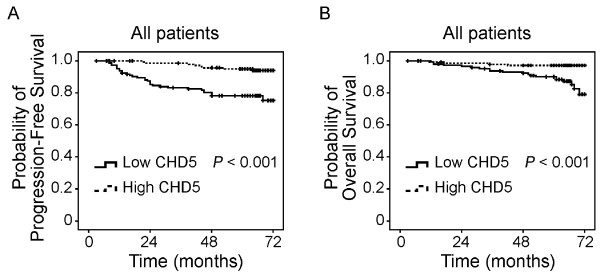
**Lack of CHD5 protein expression is significantly associated with worse patient survival**. Kaplan-Meier analysis of progression-free survival (**A**) and overall survival (**B**) in breast cancers with and without CHD5 protein expression. *P-*values were based on the log-rank test.

Among the 289 patients, 242 received adjuvant chemotherapy, 132 received endocrine treatment, and 91 received radiation therapy. The average follow-up time was 61 months (ranged from 11 to 72 months). During the follow-up, 3 patients (1%) developed local recurrence, 37 (12.8%) developed distant metastasis, and 24 (8.3%) died. Lack of CHD5 expression significantly correlated with distant metastasis (*P *< 0.001, Chi squared analysis) and death (*P *= 0.001) (Table 2). Kaplan-Meier analysis indicated that patients with no CHD5 expression had significantly shorter progression-free survival (Figure 4A) and overall survival (Figure 4B). Moreover, multivariate Cox regression analysis indicates that lack of CHD5 expression is an independent prognostic factor (Additional file 1 Table S3).

### Promoter hypermethylation of *CHD*5 in breast cancer

**Figure 5:**
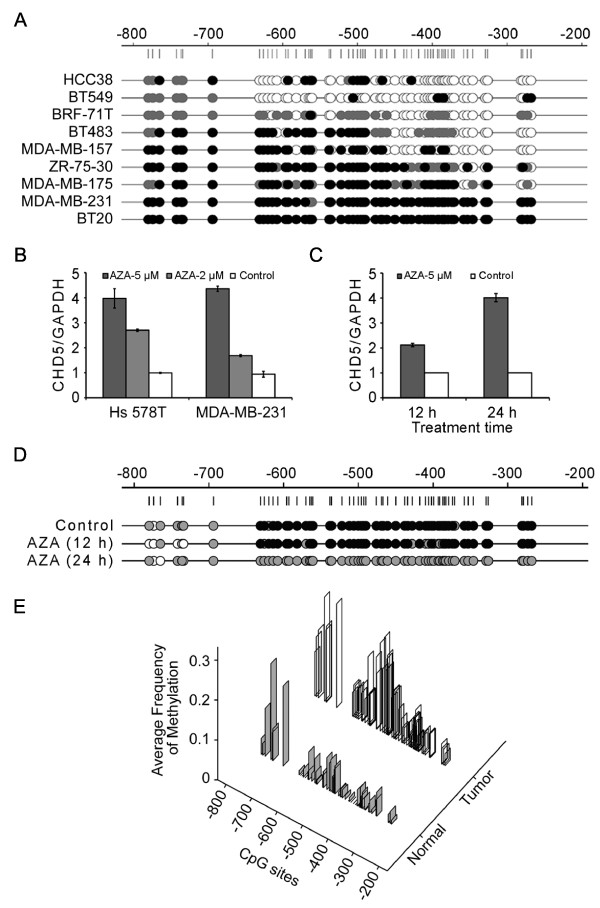
**Methylation of *CHD5 *promoter region down-regulates *CHD5 *expression in breast cancer**. (**A**) Methylation status of *CHD5 *promoter region in nine breast cancer cell lines as determined by bisulfite sequencing of cloned PCR products. The horizontal line at the top represents promoter DNA, whereas vertical lines under it indicate the relative locations of each CpG site. Methylation status of each CpG site is indicated by circles, and dark, gray and empty circles indicate high levels (methylation frequency > 50%), moderate levels (methylation frequency < 50%), and no (methylation frequency = 0) methylation, respectively. (**B, C**) Detection of *CHD5 *mRNA levels by real time PCR in Hs 578T and MDA-MB-231 cell lines treated with 2 and 5 μM 5-aza-2'-deoxycytidine (AZA) for 3 days (B) or in MDA-MB-231 cells treated with 5 μM AZA for 12 and 24 hours (h) (C). GAPDH serves as an internal control. (**D**) Methylation levels of *CHD5 *promoter in MDA-MB-231 cells treated with AZA, as detected by bisulfite treatment and sequencing of five clones from each PCR product. Empty, gray and dark circles indicate zero, one to three and four or five clones, respectively, that are methylated. (**E**) Methylation levels of *CHD5 *promoter in breast tumors (*n *= 30) and normal breast tissues (*n *= 10). The frequency of methylation (*y *axis) at each CpG site (*x *axis) was the average frequency in either the tumor or the normal group.

The methylation status of the *CHD5 *promoter region from -841 to -246, which contains 58 CpG sites and is frequently methylated in neuroblastoma cell lines [7], was determined in 9 breast cancer cell lines, 30 primary tumors, and 10 normal breast tissues. The majority of the 58 CpG sites were methylated in breast cancer cell lines BT-20, MDA-MB-231, ZR-75-30 and MDA-MB-175, while around half of the 58 CpG sites were methylated in the MDA-MB-157 and BT-483 cancer cell lines (Figure 5A). In the remaining three cancer cell lines (BRF-71T, HCC38 and BT-549), *CHD5 *promoter methylation was detectable but to a much lower extent (Figure 5A). Strikingly, the four cell lines with the most extensive methylation (BT-20, MDA-MB-231, ZR-75-30 and MDA-MB-175) had barely detectable *CHD5 *mRNA expression, and only one of the four cell lines, ZR-75-30, had a copy number loss at *CHD5 *(Additional file 1 Table S4), suggesting that promoter methylation is more often responsible for the down-regulation of *CHD5 *in some breast cancers.

To further determine whether promoter methylation is responsible for reduced *CHD5 *mRNA expression in breast cancer, we treated MDA-MB-231 and Hs 578T cell lines, both of which had little *CHD5 *mRNA expression, with the 5-aza-2'-deoxycytidine (5-aza-CdR) demethylating agent (2 and 5 μM) for three days, and examined the effect of 5-aza-CdR on *CHD5 *expression. Real-time PCR assay showed that *CHD5 *mRNA level was doubled in MDA-MB-231 cells and tripled in Hs 578T cells after treatment with 2 μM 5-aza-CdR for three days, and was quadrupled in both cell lines after treatment with 5 μM 5-aza-CdR for three days (Figure 5B), which are consistent with a previous study [9]. Treatment with 5 μM 5-aza-CdR could even increase *CHD5 *expression at 12 and 24 hours in MDA-MB-231 cells (doubled at 12 hours and quadrupled at 24 hours) (Figure 5C). As expected, sequencing of cloned bisulfite-treated DNA demonstrated a significantly decreased methylation level of *CHD5 *promoter in MDA-MB-231 cells treated with 5-aza-CdR for 24 hours (Figure 5D).

In clinical specimens, the methylation level for each CpG site was averaged in 10 normal breast tissues and 30 primary tumors and plotted against the location of each CpG site (Figure 5E). The average methylation level per CpG site was 0.1 in the tumor samples, which was significantly higher than that in the normal samples (0.036) (independent *t*- test, *P *< 0.001). The CpG sites between -626 and -399 were methylated more heavily in clinical tumors (Figure 5E).

Among the 30 primary tumors used in the methylation study, 19 had adjacent normal tissue and, therefore, were used for the detection of *CHD5 *expression by real time PCR. According to their average methylation levels, we divided the 19 tumors into two groups: one with lower levels of methylation (0 < average methylation level < 0.036, 3 cases) and the other with higher levels of methylation (average methylation level > 0.036, 16 cases). The same tumors were then divided into three groups according to the levels of CHD5 expression: low when *CHD5 *expression in a tumor was less than half of that in the matched normal tissue, moderate when the expression was more than half but less than two folds, and high when the expression was more than two folds. Notably, 12 of the 16 highly methylated tumors had low levels of *CHD5 *expression, while the 3 tumors with lower levels of methylation showed moderate or high *CHD5 *expression (*P *= 0.045, Additional file 1 Table S5). These results suggest that hypermethylation of *CHD5 *promoter region is responsible for reduced *CHD5 *mRNA expression at least in some of the breast cancers.

### CHD5 suppresses the proliferation of breast cancer cells *in vitro *and *in vivo *

**Figure 6:**
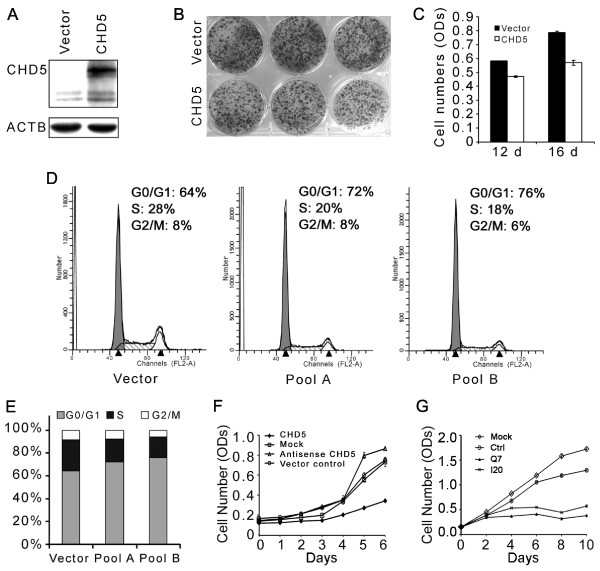
**Effect of CHD5 on cell proliferation in breast cancer cell lines**. (**A**) Confirmation of CHD5 expression in transfected MDA-MB-231 cells by Western blotting, with *β*-actin (ACTB) as a loading control. (**B**) Representative images of cells transfected with *CHD5 *or vector plasmid in six-well culture plates at 12 days of G418 selection. (**C**) Ectopic expression of *CHD5 *in MDA-MB-231 cells decreased colony formation efficiency at both 12 and 16 days of G418 selection, as detected by SRB staining. The numbers of cells are indicated by optical densities. (**D, E**) Ectopic expression of *CHD5 *increased the number of cells in the G0/G1 phase but decreased the number of cells in the S phase of the cell cycle, as determined by flow cytometer. Transfected cells were selected by G418, and pools A and B were a mixture of transfected MDA-MB-231 cells from two independent transfection and selection experiments. (**F**) Ectopic expression of *CHD5 *inhibits cell proliferation in Hs 578T cells, as measured by SRB staining assay. Parental cells without plasmid transfection (mock) and cells transfected with empty vector (vector control) and antisense *CHD5 *were used as negative controls. (**G**) Knockdown of *CHD5 *expression by siRNAs (Q7 and I20) inhibits the proliferation of T-47D cells as compared to parental cells (Mock) or siRNA control (Ctrl).

We transfected MDA-MB-231 breast cancer cells, which have a truncation mutation of *CHD5*, with *CHD5 *expression plasmid or vector control. Expression of *CHD5 *was confirmed by Western blotting (Figure 6A). After selection with G418 for 12 and 16 days, expression of *CHD5 *significantly decreased colony-forming efficiency (independent *t*-test, *P *< 0.001) (Figure 6B, C). We also examined cell cycle distribution of MDA-MB-231 cells after transfection and selection with G418. CHD5 expression increased the number of cells in G0/G1 phase while decreasing the number of cells in the S and G2/M phases (Figure 6D, E).

Cell proliferation analysis was also conducted in Hs 578T cells, in which *CHD5*-expressing plasmid or control plasmids were transfected, and the selection for positively transfected cells continued for two weeks. Compared to parental Hs 578T cells and those transfected with vector control or the plasmid expressing antisense-CHD5, proliferation activity of cells transfected with *CHD5*-expressing plasmid was significantly reduced (Figure 6F).

In the T-47D breast cancer cell line, which expresses a high level of CHD5, we knocked down CHD5 expression by RNAi, and unexpectedly detected a significant decrease in cell proliferation (Figure 6G).

**Figure 7:**
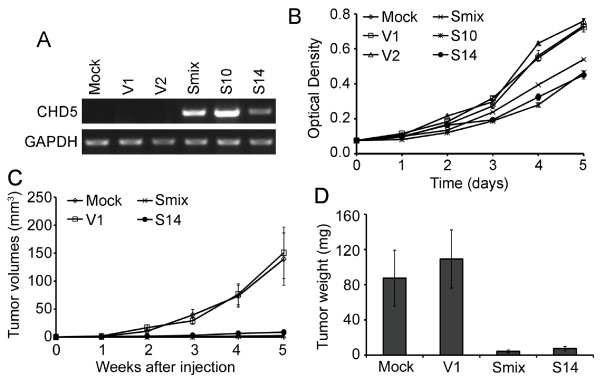
**CHD5 inhibits the growth of breast cancer cells both *in vitro *and in nude mice**. (**A**) Confirmation of *CHD5 *mRNA expression by semi-quantification RT-PCR in parental MDA-MB-231 cells, stable vector control clones (V1 and V2), and stable population (Smix) and clones (S10 and S14) of *CHD5*-transfected cells. GAPDH was used as an internal control. (**B**) Expression of *CHD5 *inhibits cell proliferation in MDA-MB-231 cells, as examined by SRB staining. (**C, D**) Expression of *CHD5 *represses tumor growth in nude mice, as indicated by tumor volumes (C) and tumor weights (D) (8 mice were used for each group).

The effect of CHD5 on cell proliferation was further tested in parental and derivative stable clones of MDA-MB-231 cells expressing varying levels of *CHD5 *(Figure 7A). Compared to parental MDA-MB-231 cells and two vector control clones (V1 and V2), clones or the population stably expressing *CHD5 *(S10, S14 and Smix) showed significantly slower cell proliferation (Figure 7B). We also examined parental MDA-MB-231 cells, the V1 vector control clone and the CHD5-expressing stable population (Smix) or clone (S14) for tumorigenesis in nude mice. While the control cell populations rapidly grew into tumors in nude mice, the CHD5-expressing cells had significantly slower growth, as indicated by tumor volumes measured at Weeks 3 to 5 and tumor weights determined at Week 5 (Figure 7C, D). These results indicate that *CHD5 *suppresses breast cancer cell growth both *in vitro *and *in vivo*.

### CHD5 suppresses invasiveness of breast cancer cells *in vitro *

**Figure 8:**
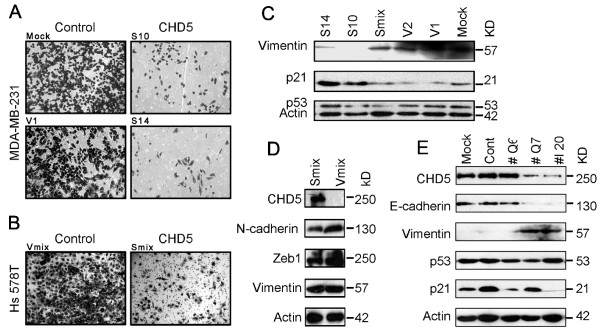
**CHD5 suppresses the invasion and the expression of EMT markers in breast cancer cells**. (**A, B**) Expression of *CHD5 *inhibits the invasion of MDA-MB-231 cells (A) and Hs 578T cells (B), as detected in the invasion assay. *CHD5 *or control is indicated at the top, and sample names are indicated at upper left. (**C-E**) Detection of different proteins by Western blotting, including vimentin, p21 and p53 in MDA-MB-231 cells without transfection (Mock) or transfected with CHD5 plasmid (Smix population and S10 and S14 clones) or control plasmid (V1 and V2 clones) (C); N-cadherin, ZEB1 and vimentin in Hs 578T cell population expressing CHD5 (Smix) or vector (Vmix) (D); and E-cadherin, vimentin, p53 and p21 in T-47D cells without transfection (Mock) or transfected with negative control siRNA (Cont) and *CHD5 *siRNAs (Q7 and I20) (E).

The invasive ability of CHD5-expressing S10 and S14 clones were significantly impaired compared to parental MDA-MB-231 cells and vector control cells (Figure 8A). In the Hs 578T cell line, cell population stably transfected with CHD5 also showed an inhibition of invasion when compared to vector-transfected cell population (Figure 8B). Consistent with a decrease in invasiveness, protein expression of vimentin, a mesenchymal cell marker, was reduced in S10 and S14 cells (Figure 8C). In addition, protein expression of N-cadherin and ZEB1, two mesenchymal cell markers not detectable in MDA-MB-231 cells, were down-regulated in CHD5-expression Hs 578T cells (Figure 8D). Furthermore, knockdown of CHD5 in T-47D cells reduced the expression of the epithelial marker E-cadherin and increased the expression of mesenchymal marker vimentin (Figure 8E), suggesting that CHD5 loss could induce EMT in CHD5-positive breast cancer cells. EMT makers N-cadherin, ZEB1 and ZEB2 were also examined in T-47D cells, but they were not detectable in this cell line. T-47D cells were not invasive in the invasion assay, and knockdown of CHD5 did not sufficiently induce invasiveness. These results suggest that in addition to modulating cell cycle, CHD5 also regulates cellular invasion by suppressing EMT in breast cancer cells.

## Discussion

Deletion of 1*p*36 is common in a variety of cancers, including breast cancer, suggesting that there is an important tumor suppressor gene in this region whose inactivation drives tumorigenesis. By analyzing mouse models generated by chromosome engineering to obtain different dosages of genomic interval D4Mit190-51, which corresponds to human 1*p*36, Bagchi *et al. *demonstrated that *CHD5 *was the leading tumor suppressor gene in this interval [2]. *CHD5 *has also been identified as a candidate tumor suppressor gene in neuroblastoma [18]. In this study, we conducted a series of studies to examine whether *CHD5 *is a tumor suppressor gene in human breast cancer. Whereas one mutation, a truncation mutation in the MDA-MB-231 breast cancer cell line, was found in *CHD5 *among the samples examined, *CHD5 *had frequent genomic deletion and promoter methylation in both breast cancer cell lines and primary tumors. Furthermore, promoter methylation correlated with transcriptional down-regulation of *CHD5 *and demethylating treatment increased *CHD5 *expression. CHD5 protein expression was also significantly reduced in primary breast tumors, and lack of CHD5 expression correlated with higher tumor grade, local recurrence, distant metastasis and worse patient survival. Functionally, CHD5 inhibited the proliferation and invasion of MDA-MB-231 and Hs 578T cells *in vitro *and tumorigenesis of MDA-MB-231 cells *in vivo *(Figures 1, 2, 3, 4, 5, 6, 7, 8). Taken together with previous findings that showed deletion of 1*p*36 in breast tumors [19,20], our results suggest that *CHD5 *is a tumor suppressor gene whose inactivation by mutation, chromosomal deletion or promoter methylation-mediated transcriptional down-regulation plays a role in the development and progression of breast cancer.

We noticed that in the T-47D breast cancer cell line, which expresses a higher level of *CHD5*, RNAi-mediated knockdown of *CHD5 *did not increase cell proliferation. Unexpectedly, *CHD5 *knockdown decreased cell proliferation. While the reason for this unexpected finding remains to be determined, one possibility is that T-47D cells have acquired molecular alterations that have reversed the function of CHD5 from a proliferation suppressor to a proliferation enhancer. Such a functional reverse has been shown previously for some molecules. For example, TGF-β is a potent tumor suppressor in early stage tumorigenesis but becomes a promoter in late stage tumor progression [21]; and deacetylation converts KLF5 from an anti-proliferative factor to a pro-proliferative in epithelial cells [22].

A tumor-specific function-altering mutation is a well-established indicator for tumor suppressor genes. In the original study that identified 122 genes with somatic mutations in breast cancer through large-scale DNA sequencing, two of the 11 (18%) primary breast cancers had a heterozygous missense mutation in *CHD5 *(133G > A, V45M; 1999A > G, R667G) [12]. In our study, none of the 38 primary tumors had somatic mutations in *CHD5*, while one of the 17 breast cancer cell lines examined had a frame shift mutation. Our results are consistent with another report in which no mutations were detected in 60 breast cancer samples while three mutations were identified in 123 ovarian cancer samples [11], indicating that, although mutation of *CHD5 *could occur in breast cancer, its frequency is rather low in primary tumors.

Among the SNPs detected in this study, some occurred frequently in cell lines but were not detected in primary tumors, including G529C (8/34 alleles or 24%) and T4715C (76%); others occurred frequently in the primary tumors but were not detected in any of the cell lines, including C2479T (30%) and G3436A (36%). These differences could be caused by differences in the ethnic background of patients whose primary tumors were used (Chinese) and patients from whom the cell lines were derived (non-Chinese). There is also a possibility that some of these SNPs contribute to successful establishment of breast cancer cell lines or even breast cancer progression, which remains to be determined.

Although somatic mutation of *CHD5 *is rare, obvious down-regulation of *CHD5 *mRNA and protein was frequent in breast cancer cell lines and primary tumors. Hemizygous deletion and promoter methylation were also common and could be responsible for transcriptional down-regulation of *CHD5 *in breast cancer. In evaluating the role of promoter methylation in the down-regulation of *CHD5*, we found that transcriptional down-regulation was significantly associated with promoter methylation and that demethylating treatment significantly increased *CHD5 *expression, indicting a role of methylation in decreasing *CHD5 *mRNA expression. These results are consistent with those from other types of tumors, such as neuroblastoma and ovarian cancer, in which frequent promoter methylation and reduced RNA expression of *CHD5 *had also been detected [7-10]. Therefore, methylation-mediated silencing, which is a common mechanism for the inactivation of tumor suppressor genes, occurs at *CHD5 *in different types of human cancers including breast cancer.

Reduced *CHD5 *mRNA expression was significantly associated with lymph node metastasis, while reduced CHD5 protein expression significantly correlated to recurrence, distant metastasis, progression-free survival and overall survival in breast cancer. Multivariate Cox regression analysis indicates that lack of CHD5 expression is an independent prognostic factor. These results are consistent with a previous report where allelic loss at 1*p *was correlated with lymph node metastasis in breast cancer [23] and that higher CHD5 expression was strongly associated with more favorable outcomes in neuroblastoma. Furthermore, the only breast cancer cell line harboring a truncated mutation at *CHD5*, MDA-MB-231, is highly metastatic. Therefore, it is possible that inactivation of CHD5 contributes to metastatic progression of breast cancer. Supporting this hypothesis, expression of *CHD5 *in the metastatic MDA-MB-231 and Hs 578T cells inhibited cellular invasiveness and down-regulated EMT markers.

Activation of the p19^Arf^/p16^Ink4a ^locus by CHD5, which activates both p19^Arf^/p53 and p16^Ink4a^/Rb signal axes, has been established as a mechanism for how CHD5 executes its tumor suppressor function. However, the three breast cancer cell lines used in our functional studies, MDA-MB-231, Hs 578T and T-47D, harbor p53 mutations [24], and no significant change in p53 expression was detected in MDA-MB-231 cells expressing ectopic *CHD5 *or T-47D cells where *CHD5 *was knocked down (Figure 8C, E). Rb expression was not changed in CHD5-expressing MDA-MB-231 cells either (data not shown). In addition, whereas the expression of p21, a target gene of p53 involved in cell cycle arrest, was increased in CHD5-expressing MDA-MB-231 cells (Figure 8C), its expression upon CHD5 knockdown in T-47D cells showed inconsistent changes, as p21 expression was decreased by one siRNA but not affected by another siRNA, although both siRNAs efficiently knocked down *CHD5 *(Figure 8E). We thus speculate that CHD5 suppresses breast carcinogenesis by a p53- and Rb-independent mechanism. In fact, other mechanisms have been suggested in other types of tumors, as *CHD5 *mutation or methylation is associated with KRAS or BRAF mutation in ovarian cancer [11], and CHD5 expression correlates with MYCN amplification in neuroblastoma [7,25].

## Conclusions

In this manuscript, we found that promoter methylation, genomic deletion and down-regulation of *CHD5 *at both RNA and protein levels are common in breast cancer, and *CHD5 *down-regulation was significantly associated with metastasis and worse patient survival in breast cancer. In addition, in the highly metastatic MDA-MB-231 breast cancer cell line, which harbors a truncating mutation, ectopic expression of *CHD5 *suppressed cell proliferation *in vitro *and tumorigenesis in nude mice by arresting the cell cycle, and inhibited cell invasion. Similar results were obtained in the Hs 578T breast cancer cell line. These results suggest that *CHD5 *is an important tumor suppressor gene that could modulate the development and progression of human breast cancer.

## Abbreviations

5-aza-CdR: 5-aza-2'-deoxycytidine; CHD: chromodomain helicase DNA binding protein; CpG: cytosine-phosphate-guanine; EMT: epithelial-mesenchymal transition; ER: estrogen receptor alpha; GAPDH: glyceraldehyde 3-phosphate dehydrogenase; HER2: human epidermal growth factor receptor 2; HGD: human genomic DNA; HMEC: human mammary epithelial cells; IHC: immunohistochemistry; LN: lymph node; M-MLV: Moloney murine leukemia virus; NuRD: nucleosome remodeling and histone deacetylation; OS: overall survival; PBS: phosphate-buffered saline; PFS: progression-free survival; PR: progesterone receptor; RT-PCR: reverse transcription polymerase chain reaction; SNP: single nucleotide polymorphism; SRB: sulforhodamine B.

## Competing interests

The authors declare that they have no competing interests.

## Authors' contributions

XW carried out most molecular, genetic and functional experiments. She also extracted DNA and RNA samples from some tissue specimens and cell lines, and drafted the manuscript. WL read tissue slides and scored CHD5 IHC staining with the guidance of LF. He also participated in animal experiments. XF conducted immunohistochemical staining of CHD5 in human tissues. DS conducted sequence data analysis and statistical analysis. LF participated in the mutation analysis of CHD5 in tumor samples and cell lines. ZZ and AL extracted DNA and RNA samples from some breast tissue specimens and cell lines. XS extracted DNA from some of the breast cancer cell lines and treated cell line DNA with sodium bisulfite for methylation analysis. LF is responsible for the collection and preparation of breast cancer tissues for analysis. ZZ and JTD designed the study, provided overall guidance and led the execution of all experiments. JTD is also responsible for the finalization of the manuscript. All authors read and approved the manuscript.

## Supplementary Material

Additional file 1 **Table S1 through S5**. Click here for file
