# Supplementary material for: Chromodomain helicase DNA binding protein 5 plays a tumor suppressor role in human breast cancer
Source: Breast Cancer Res. 2012 May 8;14(3):R73. doi: 10.1186/bcr3182 (PMC3446335; doi:10.1186/bcr3182)
Supplement: Additional file 1 — Table S1 through S5. [file bcr3182-S1.DOC]

**Additional file 1 Table S1** Association of *CHD5* mRNA expression with clinicopathological variables

|  | *CHD5* mRNA levels | |  |  |
| --- | --- | --- | --- | --- |
|  | Reduced (n)* | Normal (n)* | *Z* | *P* |
| Age (years) |  |  | -0.111 | 0.912 |
| ≤ 45 | 7 | 3 |  |  |
| > 45 | 30 | 14 |  |  |
| LN metastasis |  |  | -2.226 | 0.026 |
| Negative | 18 | 14 |  |  |
| Positive | 18 | 3 |  |  |
| Estrogen receptor |  |  | -1.741 | 0.082 |
| Negative | 12 | 10 |  |  |
| Positive | 24 | 7 |  |  |
| Progesterone receptor |  |  | -1.129 | 0.259 |
| Negative | 12 | 8 |  |  |
| Positive | 24 | 8 |  |  |
| HER2 |  |  | -1.554 | 0.12 |
| Negative | 25 | 8 |  |  |
| Positive | 11 | 9 |  |  |
| p53 |  |  | -0.366 | 0.714 |
| Negative | 21 | 9 |  |  |
| Positive | 15 | 8 |  |  |

Notes: n, number of samples; LN, lymph node; *P* values were calculated by Mann-Whitney U test. **CHD5* mRNA expression was defined as Reduced when its expression in a tumor was less than half of that in the matched normal tissue, and as Normal when its expression in a tumor was more than half of that in the matched normal tissue.

**Additional file 1 Table S2.** Expression levels of CHD5 protein among different primary tumors and normal tissues of the breast.

|  | Cases | CHD5 negative (**-)** | CHD5 positive | | |
| --- | --- | --- | --- | --- | --- |
|  |  |  | + | ++ | +++ |
| Normal tissues | 20 | 0 (0) | 3 (15.0) | 6 (30.0) | 11 (55.0) |
| Breast cancers | 289 | 144 (49.8) | 30 (10.4) | 61 (21.1) | 54 (18.7) |

Note: Numbers in parentheses are percentages of different expression groups among total cases.

**Additional file 1 Table S3.** Multivariate analyses by Cox proportional hazard ratio using backward logistic regression.

| Variable | Progression-free survival | | Overall survival | |
| --- | --- | --- | --- | --- |
|  | Hazard Ratio (95% CI) | *P* | Hazard Ratio (95% CI) | *P* |
| CHD5 | 0.278 (0.126-0.615) | 0.002 | 0.280 (0.092-0.849) | 0.024 |
| LN metastasis | 2.247 (1.018-4.961) | 0.045 | N/A | N/A |
| Histological grade | N/A | N/A | 3.104 (1.280-7.094) | 0.012 |

Notes: CI, confidence interval; N/A, data not available due to inadequate samples for analysis.

**Additional file 1 Table S4.** Summary of CHD5 copy number, methylation and expression data in breast cancer cell lines

| Cell line | *CHD5* copy  number | Methylation  status | *CHD5* mRNA  levels |
| --- | --- | --- | --- |
| ZR-75-30 | deletion | high methylation | reduced |
| BRF-71T | deletion | low methylation | reduced |
| BT549 | deletion | low methylation | reduced |
| HCC1599 | deletion | / | normal |
| HCC2218 | deletion | / | reduced |
| ZR-75-1 | Normal | / | reduced |
| BT483 | Normal | moderate methylation | reduced |
| BT474 | Normal | / | reduced |
| HCC1806 | Normal | / | normal |
| HCC202 | Normal | / | reduced |
| MDA-MB-468 | Normal | / | reduced |
| MDA-MB-134 | Normal | / | increased |
| T-47D | Normal | / | increased |
| HCC38 | Normal | low methylation | increased |
| Hs578T | Normal | / | reduced |
| MCF-7 | Normal | / | normal |
| MDA-MB-361 | Normal | / | reduced |
| DU4475 | Normal | / | reduced |
| MDA-MB-175 | Normal | high methylation | reduced |
| HCC1395 | Normal | / | reduced |
| UACC893 | Normal | / | normal |
| CAMA-1 | Normal | / | normal |
| MDA-MB-453 | Normal | / | reduced |
| MDA-MB-231 | Normal | high methylation | reduced |
| MDA-MB-157 | duplication | moderate methylation | increased |
| HCC1937 | duplication | / | increased |
| HCC70 | duplication | / | reduced |
| SW527 | duplication | / | reduced |
| MDA-MB-415 | duplication | / | reduced |
| BT20 | / | high methylation | reduced |
| HCC1500 | / | / | normal |
| HCC1143 | / | / | increased |

Note: /, not detected.

**Additional file 1 Table S5.** Correlation between *CHD5* mRNA expression and promoter methylation

| *CHD5* methylation | Total  cases | *CHD5* mRNA levels | | | *P* |
| --- | --- | --- | --- | --- | --- |
|  |  | Low (< 0.5) | Moderate (0.5-2) | High (> 2) |  |
| More methylated  (> 0.036) | 16 | 12 (75.0) | 3 (18.8) | 1 (6.3) | 0.045 |
| Less methylated  (0 - 0.036) | 3 | 0 (0) | 2 (66.7) | 1 (33.3) |  |

Notes: *P* value was calculated using the Chi-square test. Numbers in parentheses are percentages of different expression groups among total cases.
